# Optimization of Ultra-Dense Wireless Powered Networks

**DOI:** 10.3390/s21072390

**Published:** 2021-03-30

**Authors:** Panagiotis D. Diamantoulakis, Vasilis K. Papanikolaou, George K. Karagiannidis

**Affiliations:** Department of Electrical and Computer Engineering, Aristotle University of Thessaloniki, 54 124 Thessaloniki, Greece; padiaman@auth.gr (P.D.D.); vpapanikk@auth.gr (V.K.P.)

**Keywords:** wireless power transfer, internet-of-things, ultra-dense, remote radio heads, optimization

## Abstract

The internet-of-things (IoT) is expected to have a transformative impact in several different domains, including energy management in smart grids, manufacturing, transportation, smart cities and communities, smart food and farming, and healthcare. To this direction, the maintenance cost of IoT deployments has been identified as one of the main challenges, which is directly related to energy efficiency and autonomy of IoT solutions. In order to increase the energy sustainability of next-generation IoT, wireless power transfer (WPT) emerged as a promising technology; however, its effectiveness is hindered as the distance between the base station and the wireless powered IoT devices increases. To counter this effect, decentralized approaches based on the use of distributed densely deployed remote radio heads (RRHs) can be utilized to diminish the distance between the transmitting and the receiving nodes. A trade-off ensues from the use of RRHs as power beacons (PBs) or access points (APs) that enable either energy transfer during downlink or information reception during uplink, respectively. To balance this trade-off, in this work, the maximization of the ergodic rate in ultra-dense wireless powered networks is investigated. In more detail, three different protocols are introduced, optimized, and compared to each other: density splitting, time splitting, and hybrid time and density splitting, which are based on the optimization of the portion of the number of RRHs that are employed as PBs or APs at a specific time instance. Additionally, two different policies are taken into account regarding the PBs’ power constraint. The formulated problems that correspond to the combination of the proposed protocols with each of the two considered power constraint policies are optimally solved by using convex optimization tools and closed-form solutions are derived that result to useful insights. Finally, numerical results are provided, which illustrate the ergodic rate achieved by each of the proposed protocols and offer interesting conclusions regarding their comparison, which are directly linked to design guidelines and the required capital and operational expenses.

## 1. Introduction

A crucial challenge in the current and the coming wireless networks lies with the improvement of energy efficiency (EE). Specifically, the reduction of energy consumption is particularly important in the case of internet-of-things (IoT) devices, the number of which continues to soar, reaching billions of connected devices in the coming decade [1]. A pivotal issue, from which IoT devices suffer, is their limited battery lifetime, which is primarily used for communications. At the same time, their deployment makes it difficult or even impossible to replace the batteries in each device. As such, the prolongation of battery lifetime of mobile devices, i.e., sensors and actuators, has been identified as an important priority in the next-generation IoT (NGIoT) [2,3], being of paramount importance in improving autonomy, scalability, and intelligence of smart grids, manufacturing, transportation, smart cities and communities, smart food and farming, and healthcare applications. To achieve the aforementioned objectives, energy harvesting has been recognized as a promising approach for the NGIoT, which creates several non-trivial challenges at the design and optimization of appropriate communication protocols [4,5].

Wireless power transfer (WPT) has been extensively studied in the recent years as a way to alleviate the mobile devices’ energy requirements via remote charging. Ambient RF energy is highly dependent on the radio environment of operation, and as such it is plagued by a high degree of uncertainty which is detrimental to applications that need a continuous power supply. Moreover, the amount of harvested ambient energy is far from enough to provide sufficient power for most mobile devices’ operation. In this regard, dedicated power signals from wireless access points acting as power beacons can be utilized as a more effective alternative. The corresponding research has focused mainly on two different directions: simultaneous wireless information and power transfer (SWIPT) and wireless powered networks (WPNs). SWIPT focuses on the downlink of wireless networks where the transmitted signals are used both for information transmission and energy transfer. To enable this twofold use of transmitted signals, two notable examples are the power splitting and the time switching receivers. Power splitting divides a part of the received energy to be utilized for energy harvesting (EH), while time switching receivers perform EH for a fraction of the timeframe and use the rest of the time for information transmission [6,7,8]. On the other hand, in WPNs, which is the main focus of this paper, the aim is to optimize the uplink performance when power beacons are employed to provide wireless energy to mobile devices in the network. On the mobile devices’ receivers, a rectifier circuit is utilized to harvest the incoming energy and exploit it to transmit information back to the access points. This can be implemented by either using time splitting-based protocols, according to which energy harvesting and information transmission take place in different fractions of time, or by parallelizing the two procedures by using separate transceivers for each of the two procedures [9,10].

The most important challenge of WPNs is the double near-far problem, according to which the users located near the cell edge, receive a lower amount of wireless energy compared to users located near the base station, while they would also require higher transmit power in order to achieve comparable quality-of-service (QoS) during uplink [9,10,11]. This challenge can be mitigated by using decentralized approaches that reduce the distance between the remote radio heads (RRHs) and the wireless powered device [12]. These approaches result to reduce the average signal attenuation and, consequently, increase the data rates, as, due to the increase of the number of RRHs, the expected distance of a user form the nearest RRHs is reduced. This leads to ultra-dense architectures, the performance of which can be modeled and further investigated by using tools from stochastic geometry [13,14].

### 1.1. Related Work and Motivation

EH has been examined in a plethora of network scenarios. Early works on WPT have shown the possible benefits from implementing an energy harvesting architecture complementary to wireless networking [15], e.g., in cooperative communication scenarios [16]. More recently, in [17], the authors have investigated mobility scenarios in SWIPT networks in order to determine the information-energy region, which demonstrated the inherent trade-off between information reception and power transfer in SWIPT. Additionally, SWIPT networks have also been examined for device-to-device (D2D) type communications in [18], where in order to address the EE need of D2D scenarios, SWIPT was utilized through stochastic geometry to develop energy-efficient modes of operation. In [19], WPT is employed in a mobile-edge computing (MEC) architecture with the harvest-then-use protocol, so the harvested energy at the users can be exploited for offloading and for local computations. Multiple sources for EH have been examined in [20], focusing on deriving valuable statistical results for an approximate linear harvesting model and proposing optimal power transfer strategies based on these results. On top of that, in [21] WPT has been experimentally validated for the distributed antenna scenario focusing on low complexity low cost flexible deployment.

On the other hand, ultra-dense networks have been examined in the literature with the most prominent result being the increase in the network capacity. More specifically, in the pioneering work [14], the increased network density gives footing to the decoupling of downlink and uplink. Based on that, the authors were able to provide tight bounds on the uplink ergodic rate as a design tool to guarantee the quality of service (QoS) of the users. Furthermore, in [22] the authors derived closed-form expressions for the relationship between spectral efficiency (SE) and base stations’ density, showcasing the asymptotically logarithmic increase of SE with respect to the density. These results can be employed by operators in the design and planning of the network. Moreover, in [23], Park et al. proposed the use of a decoupled heterogeneous network with both mmWave and microwave bands in use, which aims to maximize the overall downlink of the network with QoS constraints imposed on the uplink. Their contributions showed that mmWave is more suitable for the downlink due to its high peak-to-average ratio, while the uplink can be better handled for the most part by sub-6 GHz frequencies.

As it has already been mentioned, ultra-dense networks reduce the distance between the access points and the devices, significantly limiting the negative effects of the double near far problem that hinders WPNs’ performance and increasing the EE [15]. More specifically, in ultra-dense networks, multiple distributed antennas are employed, some of which can act exclusively as power beacons for the mobile devices (PBs), while the rest are employed for communication purposes. In more detail, in [15] the feasibility region is investigated, which contains all feasible combinations of the network parameters under the outage constraint. Moreover, the co-existence of energy harvesting protocols with ultra-dense networking can provide increased EE, which is particularly useful for wireless powered IoT connectivity. This is showcased in [24], where the authors presented the use of SWIPT in ultra-dense networks with caching capabilities. Finally, the authors in [25] examined the required density to achieve the throughput and EH goals of the users of either a sub-6 GHz band network or a mmWave band network. In their paper, the authors determine the network behavior depending on the ratio of the number of sub-6 GHz base stations (BSs) to that of mmWave BSs. While mmWave BSs can offer higher throughput but less harvested energy, when the deployment is dense enough, the harvested energy is comparable to its sub-6 GHz counterpart. However, the maximization of the ergodic rate in ultra dense WPNs, as well as the design and comparison of appropriate protocols for this objective, have not been considered in the existing literature. In general, the use of WPT in radio access networks with distributed antennas and especially in ultra-dense networks, has not been sufficiently investigated in the existing literature.

### 1.2. Contribution

Motivated by these conclusions, the main scope of this work is the theoretical investigation of ultra-dense wireless powered networks and the introduction and comparison of appropriate protocols. Note that similar approaches and comparisons have also been presented for simpler scenarios, e.g., point-to-point or cooperative communications, which, however, do not offer any insights for ultra-dense networks. To this end, for the first time in the existing literature, we maximize the ergodic rate in an ultra-dense WPN setup, taking advantage of the close proximity of the RRHs to the IoT devices and all the available degrees-of-freedom. More specifically, a point-to-point description of the main contributions is given below:

- To balance the trade-off between the average harvested energy and the average distance between the user and the nearest RRH that is used for information transmission, we consider that the number of RRHs that are used as PBs and access points (APs) is not fixed, but is optimized based on the system’s parameters with the aim to maximize the ergodic rate. To this end, three different protocols are proposed and compared to each other: *time splitting*, *density splitting*, and *hybrid time and density splitting*. Density splitting is based on the optimal selection of the portion of the RRHs that are used as PBs or APs, enabling the simultaneous energy harvesting and information transmission. On the other hand, according to time splitting, it is assumed that all the RRHs are used either as PBs or as APs for a portion of time which is subject to optimization. Furthermore, in hybrid time and density splitting, the time is split in two phases with the first one being dedicated solely to energy harvesting and the second being used for both energy harvesting and information transmission.
- Two different policies are taken into account regarding the power constraints, according to which the transmit power of each PB is limited by a peak power constraint or a constraint is imposed solely to the average transmit power of the PBs. Note that the investigation of both policies is useful as they correspond to different design limitations. The peak average constraint is imposed due to safety issues and regulations or hardware limitations, while the average power constraint corresponds to limitations to the consumed energy, which is also an important metric in future wireless networks. The formulated problems that correspond to the combination of the proposed strategies with each of the two considered power constraints, are optimally solved by using convex optimization tools and closed-form solutions are derived that provide useful insights.
- Numerical results illustrate that time splitting outperforms density splitting when an average power constraint is imposed or when the transmit power of the power beacons and the RRHs density are relatively small. Moreover, it is shown that the performance of hybrid time and density splitting for specific system’s parameters can be achieved by using either time or density splitting.

## 2. System Model

**Figure 1:**
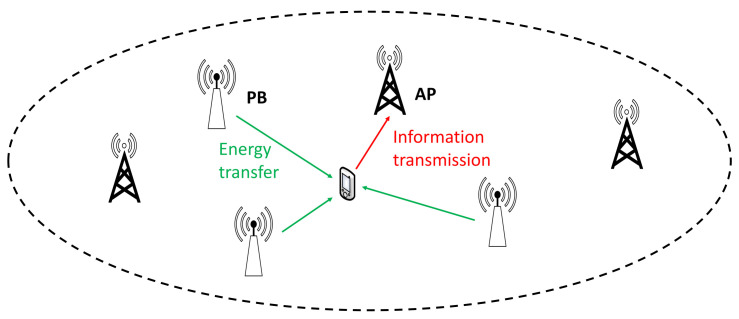
Energy transfer and information transmission in ultra-dense wireless powered networks (WPNs).

A wireless network is considered that consists of a set of densely deployed RRHs with their position being generated according to a homogeneous Poisson point process (PPP) of density λ (RRHss/m${}^{2}$), as in Figure 1. It is assumed that the RRHs are equipped with a single antenna, while each user employs two antennas: one for energy harvesting and one for information transmission, respectively [15,26]. The use of two antennas is motivated by the fact that the antennas that are used for information reception and energy harvesting need to have different sensitivity [27]. Furthermore, this setup is realistic, as, due to electronics miniaturization, even mobile and IoT devices can be equipped with more than one antennas [28]. It is further assumed that part of the RRHs are used as PBs in order to transfer energy to the considered user, with the rest being used as APs, receiving the user’s messages. Similarly to the work in [14], the considered system model corresponds to an interference-free multi-user scenario, which is practical due to the spatial separation of the energy harvesting users and the use of orthogonal multiple access schemes, according to which nodes that would otherwise interfere with each other communicate by using orthogonal resource blocks, i.e., different channels at the frequency, time, or code domain. Moreover, each user is served by its nearest AP during uplink and the corresponding ergodic rate is assumed to be independent to the user’s position due to the assumption that the RRHs are deployed according to a Poisson point process with density λ. Furthermore, it is considered that all RRHs can operate both as APs and as PBs. Moreover, in a specific time instance, part of the available RRHs can operate as APs and the other as PBs, with their positions being generated according to a homogeneous PPP of density $\lambda_{\mathrm{ap}}$ (APs/m${}^{2}$) and $\lambda_{\mathrm{pb}}$ (PBs/m${}^{2}$), respectively, with

(1) $$\lambda_{\mathrm{ap}}+\lambda_{\mathrm{pb}}=\lambda .$$

Hereinafter, it is assumed that the users are equipped with an energy storage device, e.g., a battery, which is only charged by wireless power transfer. Moreover, in order to guarantee mobile devices’ autonomy, it is further considered that the average consumed power is equal to the average harvested energy. Furthermore, to avoid unnecessary notational complexity, it is assumed that the energy is solely consumed for information transmission, which is a realistic assumption for internet-of-things devices.

### 2.1. Average Harvested Power

Assuming that the PBs transmit power in an omnidirectional way and, thus, all of them contribute to WPT, the harvested power is given by

(2) $$P_{\mathrm{har}}=\eta E\left[P_{r}\right],$$

where 0≤η≤1 is the energy harvesting efficiency and $P_{r}$ is the power received by the user from the *N* PBs that contribute more to the energy harvesting due to their shorter distance to the user, which is given by

(3) $$P_{r}=p_{d}\sum_{i=1}^{N}\frac{h_{i}}{d_{i}^{\beta }},$$

with $p_{d}=\frac{P_{d}}{L_{\mathrm{ref}}}$. Furthermore, $P_{d}$ and $L_{\mathrm{ref}}$ denote the transmit power and the equivalent path-loss at a reference distance of 1 m, respectively. Moreover, $h_{i}$, $d_{i}$, and β are the channel power gain coefficient due to fading, the distance between the user and its *i*-st nearest PB, and the path-loss exponent, respectively. Furthermore, assuming Rayleigh fading, $h_{i}$ follows the exponential distribution with parameter equal to 1. Based on the work in [13] and by considering that $E[h_{i}]=1,\forall i$, with E[·] denoting the expected value of [·], the average received power is lower-bounded by [13]

(4) $$E\left[P_{r}\right]\geq p_{d}{\left(\lambda_{\mathrm{pb}}\pi \right)}^{\frac{\beta }{2}}\sum_{n=1}^{N}\frac{\Gamma (n)}{\Gamma \left(n+\frac{\beta }{2}\right)},$$

where Γ(·) is the gamma function, and *N* is the number of the nearest PBs that are considered to have a meaningful contribution to energy transfer. Therefore, a tractable lower bound of the average harvested power is given by

(5) $$P_{\mathrm{har}}=\eta p_{d}{\left(\lambda_{\mathrm{pb}}\pi \right)}^{\frac{\beta }{2}}\sum_{n=1}^{N}\frac{\Gamma (n)}{\Gamma \left(n+\frac{\beta }{2}\right)}.$$

### 2.2. Ergodic Rate

Similarly to the work in [14], it is considered that during uplink each wireless powered device is served by its nearest AP. The capacity between a user at distance $d_{0}$ from the closest AP is given by [14]

(6) $$R=\log_{2}\left(1+\frac{\gamma_{u}h_{0}}{d_{0}^{\beta }}\right),$$

where $P_{u}$, $\sigma^{2}$ is the noise power, and $\gamma_{u}$ are the user’s transmit power, the noise power, and the SNR at the reference distance, respectively, with

(7) $$\gamma_{u}=\frac{P_{u}}{\sigma^{2}L_{\mathrm{ref}}}.$$

Moreover, $h_{0}$ is the fading coefficient which, similarly to $h_{i}$ also follows the exponential distribution with parameter equal to 1. Moreover, as it has been proved in [14], by using the Jensen’s inequality a very tight lower bound of the ergodic capacity is given by

(8) $$R=\log_{2}\left(1+\gamma_{u}{\left(\lambda_{\mathrm{ap}}\pi \right)}^{\frac{\beta }{2}}\rho \exp\left(\beta \frac{\psi }{2}\right)\right),$$

where

(9) ρ=exp(−ψ),

with ψ≈0.577 being the Euler–Mascheroni constant.

## 3. Density Splitting

The use of RRHs either as APs or PBs creates an interesting trade-off between the average harvested energy and the average distance between the user and the shortest AP. To this end, in this section the lower bound of the ergodic rate is maximized by optimizing $\lambda_{\mathrm{ap}}$ and $\lambda_{pb}$. Let θ denote the portion of RRHs that used as information receivers. In this case, a thinning is performed on the PPP according to θ resulting in two independent PPPs that describe the APs and the PBs, respectively [29]. This can easily be implemented assigning to each RRH the AP or PB operation mode with probability θ or 1−θ, respectively. This procedure follows the optimal selection of θ, which can be performed by each RRH or a central unit by using the optimal solutions that will be derived in this section. Accordingly, the density of RRHs that operate as APs is

(10) $$\lambda_{\mathrm{ap}}=\theta \lambda ,$$

while the density of PBs that operate as PBs is

(11) $$\lambda_{\mathrm{pb}}=\left(1-\theta \right)\lambda .$$

Based on the above, the lower bound of the ergodic rate can be written as

(12) $$R_{\mathrm{DS}}=\log_{2}\left(1+\gamma_{u}{\left(\theta \lambda \pi \right)}^{\frac{\beta }{2}}\rho \exp\left(\beta \frac{\psi }{2}\right)\right),$$

while the average harvested power can be expressed as

(13) $$P_{\mathrm{har},\mathrm{DS}}=\eta p_{d}{\left((1-\theta )\lambda \pi \right)}^{\frac{\beta }{2}}\sum_{n=1}^{N}\frac{\Gamma (n)}{\Gamma \left(n+\frac{\beta }{2}\right)},$$

Furthermore, due to the assumption that solely the harvested energy can be used for information transmission, the user’s transmit power can be written as

(14) $$P_{u}\leq P_{\mathrm{har},\mathrm{DS}}.$$

Finally, the corresponding optimization problem can be formulated as

(15) $$\begin{array}{cc} \max_{\theta ,P_{u}}\; & R_{\mathrm{DS}}\\ s.t.\; & C_{1}:P_{u}\leq P_{\mathrm{har},\mathrm{DS}},\\ \; & C_{2}:0\leq \theta \leq 1. \end{array}$$

In the following subsections, the aforementioned problem is optimally solved considering two different policies regarding the transmit power of each PB.

### 3.1. Peak Power Constraint

According to this policy, the transmit power of each PB is fixed and does not depend on the number of power beacons, i.e.,

(16) $$P_{d}=P_{d,\mathrm{ref}},$$

where $P_{d,\mathrm{ref}}$ is fixed for each PB.

Taking into account that $P_{\mathrm{har}}$ is an increasing function with respect to $P_{u}$, it holds that

(17) $$P_{u}^{*}=P_{\mathrm{har},\mathrm{DS}},$$

where ${\left(\cdot \right)}^{*}$ denotes optimality. Thus, the optimization problem in (15) becomes equivalent to

(18) $$\begin{array}{cc} \max_{\theta }\; & R_{\mathrm{DS},1}\\ s.t.\; & C_{1}:0\leq \theta \leq 1, \end{array}$$

where

(19) $$R_{\mathrm{DS},1}=\log_{2}\left(1+\frac{\eta P_{d,\mathrm{ref}}{\left(\lambda \pi \right)}^{\beta }{\left((1-\theta )\theta \right)}^{\frac{\beta }{2}}\rho \exp\left(\beta \frac{\psi }{2}\right)\sum_{n=1}^{N}\frac{\Gamma (n)}{\Gamma \left(n+\frac{\beta }{2}\right)}}{\sigma^{2}L_{\mathrm{ref}}^{2}}\right).$$

In turn, the optimization problem in (18) can be equivalently expressed as

(20) $$\begin{array}{cc} \max_{\theta }\; & (1-\theta )\theta \\ s.t.\; & C_{1}:0\leq \theta \leq 1. \end{array}$$

Considering that the objective function of (20) is concave and by taking its first derivative equal to zero, it is proved that the optimal value of θ for this case is

(21) $$\theta^{*}=\frac{1}{2}.$$

This is a very interesting result, as it shows that when the PBs are subject to peak power constraints, in order to maximize the ergodic rate, half of the RRHs should be used as PBs, independently of β, λ, and $P_{d,\mathrm{ref}}$.

### 3.2. Average Power Constraint

According to this policy, solely a sum constraint is enforced to the transmit power of the PBs, i.e., the transmit power of each of them depends on their number. Thus, in this case $P_{d}$ can be written as

(22) $$P_{d}=\frac{P_{d,\mathrm{ref}}}{\theta },$$

such as the number of power beacons increases their transmit power reduces. Note that based on (22), if all RRHs are used as PBs, the transmit power of each of them is equal to $P_{d,\mathrm{ref}}$.

Therefore, in this case the optimization problem in (15) can be written as

(23) $$\begin{array}{cc} \max_{\theta }\; & R_{\mathrm{DS},2}\\ s.t.\; & C_{1}:0\leq \theta \leq 1, \end{array}$$

with

(24) $$R_{\mathrm{DS},2}=\log_{2}\left(1+\frac{\eta P_{d,\mathrm{ref}}{\left(\lambda \pi \right)}^{\beta }{\left((1-\theta )\theta \right)}^{\frac{\beta }{2}}\rho \exp\left(\beta \frac{\psi }{2}\right)\sum_{n=1}^{N}\frac{\Gamma (n)}{\Gamma \left(n+\frac{\beta }{2}\right)}}{\theta \sigma^{2}L_{\mathrm{ref}}^{2}}\right).$$

The optimization problem in (23) is equivalent to

(25) $$\begin{array}{cc} \max_{\theta ,f,P}\; & {\left(1-\theta \right)}^{\frac{\beta }{2}}\theta^{\frac{\beta }{2}}\theta^{-1}\\ s.t.\; & C_{1}:0\leq \theta \leq 1, \end{array}$$

which in turn can be equivalently expressed as

(26) $$\begin{array}{cc} \max_{\theta ,f,P}\; & \frac{\beta }{2}\log\left(1-\theta \right)+\left(\frac{\beta }{2}-1\right)\log\left(\theta \right)\\ s.t.\; & C_{1}:0\leq \theta \leq 1. \end{array}$$

The objective function of (26) is concave as a sum of concave functions and its value as θ→0 and θ→1 is approaches to −∞. Thus, the optimal solution of θ is given by

(27) $$\frac{d\left(\frac{\beta }{2}\log\left(1-\theta \right)+\left(\frac{\beta }{2}-1\right)\log\left(\theta \right)\right)}{d\theta }=0,$$

from which it is derived that when β≠2, it holds that

(28) $$\theta^{*}=\frac{\beta -2}{2(\beta -1)},$$

while when β=2, $\theta^{*}\to 0$. Thus, it becomes apparent that $\theta^{*}<0.5$. Furthermore, it has been proved that when the total consumed energy is fixed, the optimal value of θ is lower compared to the case that the transmit power per power beacon is fixed.

## 4. Time Splitting

**Figure 2:**
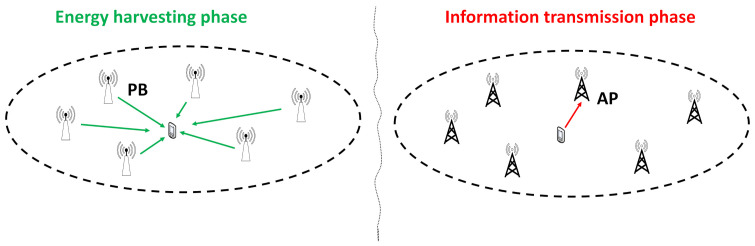
Time splitting in ultra-dense WPNs.

In this section, an alternative strategy is proposed to balance the trade-off between the harvested energy and the average distance between the user and the serving AP that exploits that time domain. This strategy is based on the use of two phases, as it is shown in Figure 2. More specifically, all the RRHs are used as PBs for the portion of time 1−τ during which the user harvests energy, while all RRHs are used as APs for the portion of time 0≤τ≤1, during which the user transmits information. Therefore, the ergodic rate for this strategy can be written as

(29) $$R_{\mathrm{TS}}=\tau \log_{2}\left(1+\gamma_{u}{\left(\lambda \pi \right)}^{\frac{\beta }{2}}\rho \exp\left(\beta \frac{\psi }{2}\right)\right),$$

while the average harvested power can be expressed as

(30) $$P_{\mathrm{har},TS}=\left(1-\tau \right)\eta p_{d}{\left(\lambda \pi \right)}^{\frac{\beta }{2}}\sum_{n=1}^{N}\frac{\Gamma (n)}{\Gamma \left(n+\frac{\beta }{2}\right)}.$$

Furthermore, due to the assumption that solely the harvested energy can be used for information transmission, while solely the portion τ of time is used for information transmission, the user’s transmit power is constrained by

(31) $$P_{u}\leq \frac{P_{\mathrm{har},\mathrm{TS}}}{\tau }.$$

Finally, the corresponding ergodic rate maximization problem can be formulated as

(32) $$\begin{array}{cc} \max_{\tau ,P_{u}}\; & R_{\mathrm{TS}}\\ s.t.\; & C_{1}:P_{u}\leq P_{\mathrm{har},TS},\\ \; & C_{2}:0\leq \tau \leq 1. \end{array}$$

Next, similarly to the case the DS strategy is used, two policies are considered regarding the transmit power of the PBs.

### 4.1. Peak Power Constraint

When this policy is adopted, the transmit power of each PB limited by a peak power constraint and remains constant regardless the time that is allocated to energy harvesting, i.e., it is given by

(33) $$P_{d}=P_{d,\mathrm{ref}}.$$

Furthermore, for the solution that maximizes the ergodic rate, (31) holds with equality. Therefore, the ergodic rate maximization problem can be written as

(34) $$\begin{array}{cc} \max_{\tau }\; & R_{\mathrm{TS},1}\\ s.t.\; & C_{1}:0\leq \tau \leq 1, \end{array}$$

where

(35) $$R_{\mathrm{TS},1}=\tau \log_{2}\left(1+\frac{\left(1-\tau \right)\eta P_{d,\mathrm{ref}}{\left(\lambda \pi \right)}^{\beta }\rho \exp\left(\beta \frac{\psi }{2}\right)\sum_{n=1}^{N}\frac{\Gamma (n)}{\Gamma \left(n+\frac{\beta }{2}\right)}}{\tau \sigma^{2}L_{\mathrm{ref}}^{2}}\right).$$

Note that $R_{\mathrm{TS},1}$ becomes equal to zero for τ=0 and τ=1. Moreover, for its second derivative, it holds that

(36) $$\frac{dR_{\mathrm{TS},1}^{2}}{d^{2}\tau }=-\frac{1}{\tau {\left(-1+\tau \right)}^{2}},$$

which is negative ∀τ>0. Therefore, $R_{\mathrm{TS},1}$ is concave for 0<τ≤1. Thus, its optimal value is given by

(37) $$\frac{dR_{\mathrm{TS},1}}{d\tau }=0,$$

which yields

(38) $$\log\left(a(\frac{1}{\tau }-1)\right)=\frac{1}{\tau -1},$$

where

(39) $$a=\frac{\eta P_{d,\mathrm{ref}}{\left(\lambda \pi \right)}^{\beta }\rho \exp\left(\beta \frac{\psi }{2}\right)\sum_{n=1}^{N}\frac{\Gamma (n)}{\Gamma \left(n+\frac{\beta }{2}\right)}}{\sigma^{2}L_{\mathrm{ref}}^{2}}.$$

By solving (38), the optimal value of τ is given in closed-form by

(40) $$\tau^{*}=\frac{a}{a+\frac{a-1}{W(\frac{a-1}{e})}-1},$$

where W(x) returns the principal branch of the Lambert W function, also called omega function or product logarithm. This function is defined as the set of solutions of the equation $x=W\left(x\right)e^{W(x)}$ [30].

### 4.2. Average Power Constraint

This policy is based on the assumption that the transmit power of each PB is increased as the portion of time that is allocated to energy harvesting reduces, such as that the sum transmit power considering both phases does not depend on the time that is allocated to energy harvesting, i.e.,

(41) $$P_{d}=\frac{P_{d,\mathrm{ref}}}{1-\tau }.$$

Thus, the ergodic rate can be written as

(42) $$R_{\mathrm{TS},2}=\tau \log_{2}\left(1+\frac{\eta P_{d,\mathrm{ref}}{\left(\lambda \pi \right)}^{\beta }\rho \exp\left(\beta \frac{\psi }{2}\right)\sum_{n=1}^{N}\frac{\Gamma (n)}{\Gamma \left(n+\frac{\beta }{2}\right)}}{\tau \sigma^{2}L_{\mathrm{ref}}^{2}}\right),$$

while the corresponding ergodic maximization problem can be formulated as

(43) $$\begin{array}{cc} \max_{\tau }\; & R_{\mathrm{TS},2}\\ s.t.\; & C_{1}:0\leq \tau \leq 1. \end{array}$$

It can easily be proved that $R_{\mathrm{TS},2}$ is an increasing function with respect to τ, and thus its optimal value is

(44) $$\tau^{*}\to 1.$$

## 5. Hybrid Time and Density Splitting

In this section, a novel strategy is proposed that can be seen as a generalization of the aforementioned two strategies. More specifically, the time is split in two phases with the first one being dedicated solely to energy harvesting and the second being used for both energy harvesting and information transmission. To this end, for the portion of time (1−τ) that is dedicated to energy harvesting all RRHs operate as PBs, while for the rest time only a subset of RRHs operate as PBs and 0≤θ≤1. Hereinafter, in this subsection θ will solely refer to the second phase. Note that both the values of τ and θ are subject to optimization. Moreover, for τ=0 and θ=1, this protocol corresponds to DS and TS, respectively. Furthermore, it is noted that the investigation of this strategy is meaningful only if a peak power constraint is imposed to each PB; otherwise, it can easily be shown that its performance coincides with TS. Thus, hereinafter, it is assumed that the transmit power of each PB, when hybrid time and density splitting is used, is given by

(45) $$P_{d}=P_{d,\mathrm{ref}}.$$

Taking into account the above, as well as (5), the harvested power considering both phases can be expressed as

(46) $$P_{\mathrm{har}},H=\eta P_{d,\mathrm{ref}}\left(\left(1-\tau \right){\left(\lambda \pi \right)}^{\frac{\beta }{2}}+\tau {\left((1-\theta )\lambda \pi \right)}^{\frac{\beta }{2}}\right)\sum_{n=1}^{N}\frac{\Gamma (n)}{\Gamma \left(n+\frac{\beta }{2}\right)},$$

while the ergodic rate is given by

(47) $$R_{H}=\tau \log_{2}\left(1+\gamma_{u}{\left(\theta \lambda \pi \right)}^{\frac{\beta }{2}}\rho \exp\left(\beta \frac{\psi }{2}\right)\right).$$

Furthermore, due to the assumption that solely the harvested energy can be used for information transmission, the user’s transmit power can be written as

(48) $$P_{u}\leq \frac{P_{\mathrm{har},H}}{\tau }.$$

Similarly to the optimization of DS and TS strategies, as $R_{G}$ is an increasing function with respect to $P_{u}$, it is considered that (48) holds with equality. Therefore, the ergodic rate maximization problem can be formulated as

(49) $$\begin{array}{cc} \max_{\tau ,\theta }\; & R_{H}\\ s.t.\; & C_{1}:0\leq \tau \leq 1,\\ \; & C_{2}:0\leq \theta \leq 1, \end{array}$$

where

(50) $$R_{H}=\tau \log_{2}\left(1+\frac{P_{\mathrm{har}},H{\left(\theta \lambda \pi \right)}^{\frac{\beta }{2}}\rho \exp\left(\beta \frac{\psi }{2}\right)}{\tau \sigma^{2}L_{\mathrm{ref}}^{2}}\right)$$

The optimization problem in (49) is non-convex and, thus, difficult to solve. To this end, a one-dimensional search is used to find the optimal value of θ. For a given value of θ, the optimization problem in (49) can be written as

(51) $$\begin{array}{cc} \max_{\tau }\; & {\tilde{R}}_{H}\\ s.t.\; & C_{1}:0\leq \tau \leq 1, \end{array}$$

where

(52) $${\tilde{R}}_{H}=\tau \log_{2}\left(1+\frac{\tilde{a}\left(1-\tau \right)}{\tau }+b\right),$$

with $\tilde{a}$ and β being given by

(53) $$\tilde{a}=\frac{\eta P_{d,\mathrm{ref}}{\left(\lambda \pi \right)}^{\beta }\theta^{\frac{\beta }{2}}\rho \exp\left(\beta \frac{\psi }{2}\right)\sum_{n=1}^{N}\frac{\Gamma (n)}{\Gamma \left(n+\frac{\beta }{2}\right)}}{\sigma^{2}L_{\mathrm{ref}}^{2}}$$

and

(54) $$b=\tilde{a}{\left(1-\theta \right)}^{\frac{\beta }{2}},$$

respectively. Taking into account the second derivative of ${\tilde{R}}_{G}$, for which it holds that

(55) $$\frac{d{\tilde{R}}_{H}^{2}}{d^{2}\tau }=-\frac{{\tilde{a}}^{2}}{\tau {\left(\tilde{a}-\tilde{a}\tau +b\tau \right)}^{2}}<0,\forall \tau >0,$$

it is proved that ${\tilde{R}}_{H}$ is concave with respect to τ. Furthermore, by setting

(56) $$\frac{d{\tilde{R}}_{H}}{d\tau }=0,$$

the following equation is derived:

(57) $$\log\left(b+\tilde{a}\left(\frac{1}{\tau }-1\right)\right)=\frac{\tilde{a}}{\tilde{a}-\tilde{a}\tau +b\tau }.$$

By solving (57) and taking into account that τ<1, the optimal value of τ is given by

(58) $$\tau^{*}=\min\left(\frac{\tilde{a}}{\tilde{a}-b+\frac{\tilde{a}-b-1}{W(\frac{\tilde{a}-b-1}{e})}+1},1\right),$$

where min(x,y) denotes the minimum value between *x* and *y*.

## 6. Numerical Results

For the demonstration of the performance that is achieved by the considered protocols, an ultra-dense wireless network has been considered with the positions of the RRHs being generated according to a homogeneous Poisson point process (PPP) of density λ (RRHss/m${}^{2}$). To compare the performance of the considered protocols, different scenarios for the PBs’ transmit power, the RRHs density, and the path-loss factor are examined, which are defined separately for each figure, assuming that η=0.8, $\sigma^{2}=-104$ dBm, and $L_{\mathrm{ref}}=20$ dB [14]. The number of RRHs that has a meaningful impact to energy harvesting, i.e., *N*, was considered to be equal to 100. The illustrated results have been produced by using the derived closed-form solutions, i.e., (21), (28), (40), (44), and (58), as well as the corresponding expressions for the ergodic rate. Note that the RRHs density and the PBs’ transmit power directly affect the required capital and operational expenses for the implementation of the considered framework.

**Figure 3:**
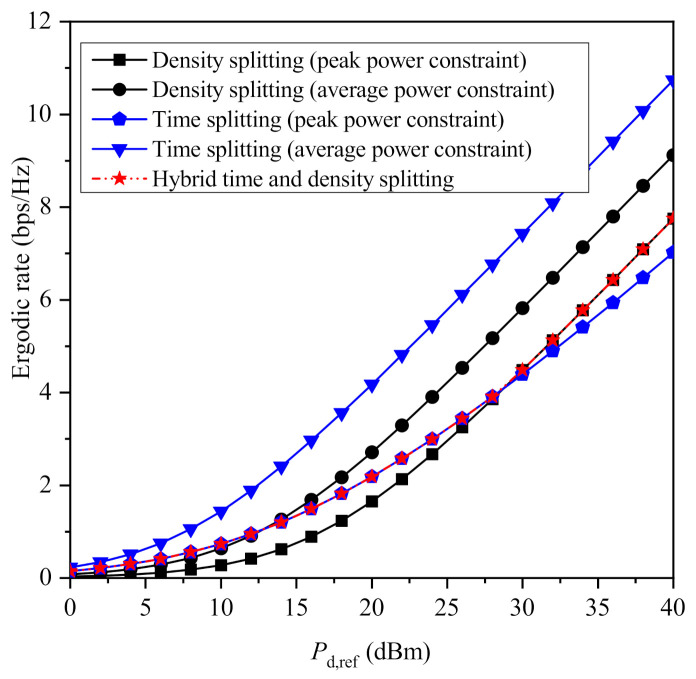
Maximized ergordic rate versus $P_{d,\mathrm{ref}}$ for β=3.

**Figure 4:**
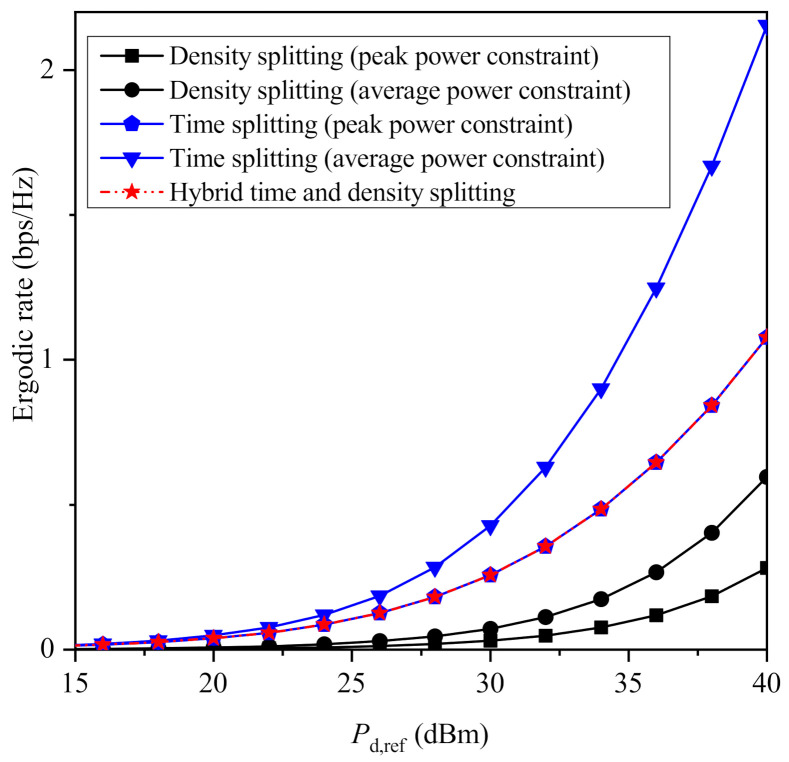
Maximized ergordic rate versus $P_{d,\mathrm{ref}}$ for β=4.

In Figure 3 and Figure 4, the maximized ergodic rate achieved by all the proposed protocols is plotted versus $P_{d,\mathrm{ref}}$ for β=3 and β=4, respectively, assuming that $\lambda =10^{-3}$ (RRHs/m${}^{2}$). An interesting observation is that when a peak power constraint is assumed for each PB, neither time splitting nor density splitting outperforms each other for all values of the system’s parameters. In more detail, for β=3, time splitting achieves higher performance to density splitting for lower values of $P_{d,\mathrm{ref}}$, while the opposite occurs for higher values of $P_{d,\mathrm{ref}}$. This is because when the average transmit power is relatively low, mitigating the path-loss is more important than increasing the total time that is used for both information transmission and energy harvesting. On the other hand, as it was expected, hybrid time and density splitting achieves the maximum performance when an individual power constraint is imposed to each PB. However, note that its performance coincides with either time splitting or density splitting, indicating that one of these two protocols achieves the maximum performance of the hybrid protocol under a specific setup. Furthermore, it is remarkable that when the path-loss factor increases, time splitting outperforms density splitting for the whole of the considered values for $P_{d,\mathrm{ref}}$. Another interesting observation is that when an average power constraint is imposed, time splitting clearly outperforms density splitting for all the considered values of $P_{d,\mathrm{ref}}$, regardless of the value of β. Moreover, as it was expected, both protocols improve when an average power constraint is imposed instead of a peak power constraint. This is due to the fact the enforcement of an average power constraint increases the amount of energy that can be consumed by the PBs, which makes the comparison between the different policies of the same protocol unfair. On the other hand, the comparison between time splitting with average power constraint and the corresponding density splitting scheme is meaningful, because both schemes lead to exactly the same energy consumption. Interestingly, this comparison leads to the noticeable conclusion that time splitting achieves higher performance compared to density splitting in terms of ergodic rate for all values of $P_{d,\mathrm{ref}}$ when an average power constraint is considered.

**Figure 5:**
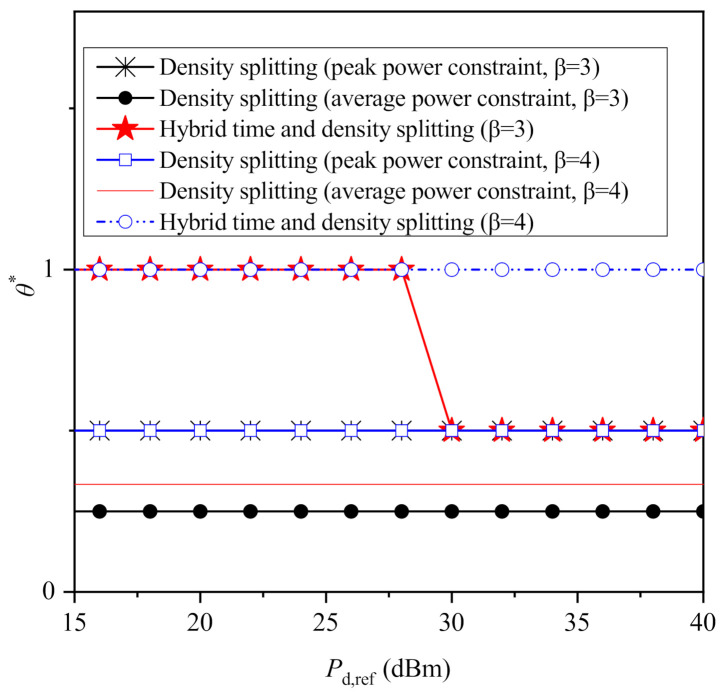
Optimized θ versus $P_{d,\mathrm{ref}}$ for β=3 and β=4.

**Figure 6:**
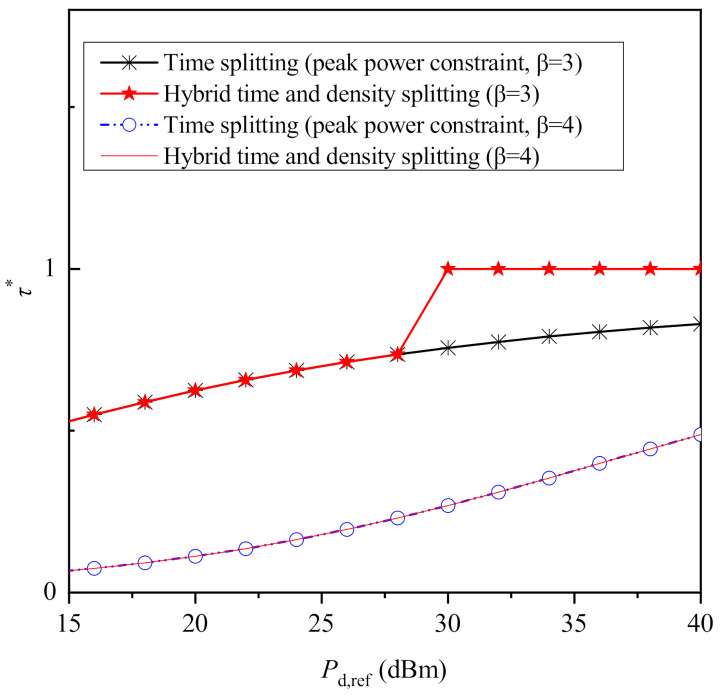
Optimized τ versus $P_{d,\mathrm{ref}}$ for β=3 and β=4.

To give further insight on the behavior of the considered protocols with the increase of the PBs’ transmit power as presented in Figure 3 and Figure 4, the optimal values of θ and τ are illustrated in Figure 5 and Figure 6 for both β=3 and β=4. As it was expected, taking into account the closed-form solutions for θ its optimal value is always equal to 0.5 when a peak power constraint is imposed to each PB. However, when an average power constraint is imposed, the optimal value of θ becomes lower to 0.5 and increases solely with the increase of β, receiving the values 1/4 and 1/3 for β=3 and β=4, respectively. On the other hand, the optimal value of θ for the hybrid protocol receives a value different to 1 only for β=3 and for higher values of $P_{d,\mathrm{ref}}$, i.e., when its performance coincides with the performance of density splitting. Next, regarding time splitting, as it is observed through Figure 6, when peak power constraints are imposed, the time that is allocated to information transmission increases with the increase of $P_{d,\mathrm{ref}}$. On the other hand, note that for the case of average power constraint, the optimal value of τ is omitted, because it has already been shown $\tau^{*}\to 1$. Moreover, it is observed that for β=3 and for $P_{d,\mathrm{ref}}<30$ dBm or for β=4, the optimal value of the hybrid protocol is the same with the optimal one for the case of time splitting with peak power constraints. On the other hand, for β=3 and for $P_{d,\mathrm{ref}}\geq 30$ dBm the optimal value of τ for the hybrid protocol becomes equal to 1, because in this case the maximized performance of the hybrid protocol is achieved by using density splitting, and, thus, no fraction of time is dedicated solely to energy harvesting.

**Figure 7:**
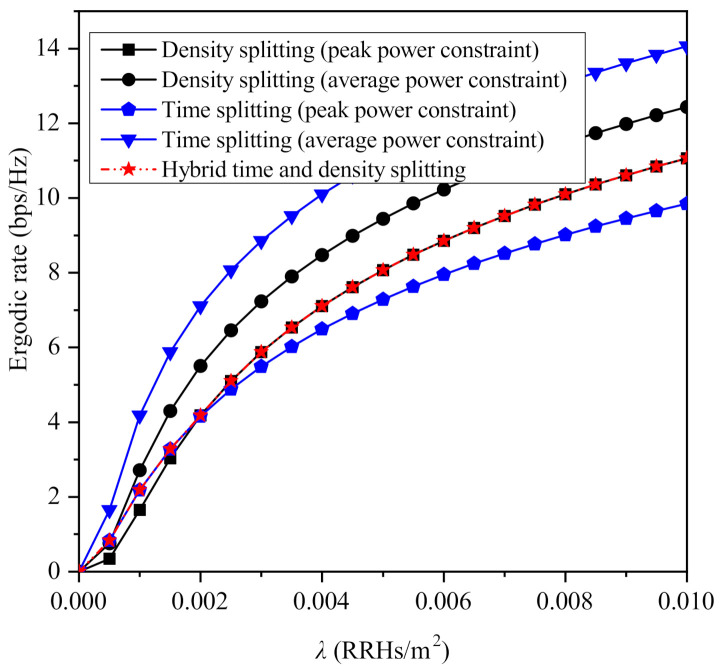
Maximized ergordic rate versus the density λ for β=3.

**Figure 8:**
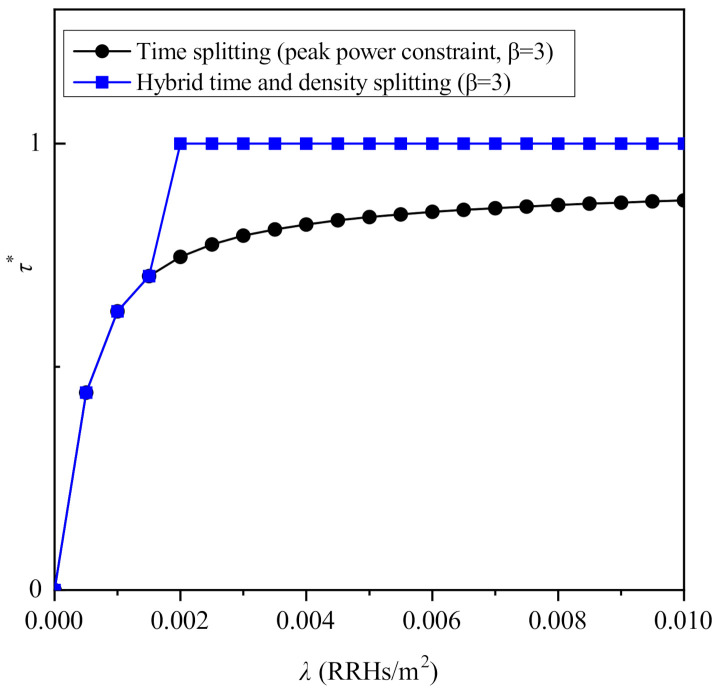
Optimized τ versus the density λ for β=3.

Moreover, Figure 7 focuses on the impact of the RRHs’ density on the maximized ergodic rate that is achieved by the considered protocols, for β=3 and $P_{d,\mathrm{ref}}=20$ dBm. As it is observed, although the ergodic rate increases with the increase of density for all the considered protocols, their performance is saturated after a certain value of λ. Regarding the case of peak power constraints, it is shown that for relatively lower values of λ, time splitting outperforms density splitting, while the opposite occurs for higher values of θ. This is because when λ is relatively low, mitigating the path-loss is more important than extending the time that is used for information transmission and energy harvesting. Consequently, it is preferable to maximize the density of both PBs and APs at the corresponding phases, despite eliminating the opportunity to simultaneously transmit information and harvest energy. Furthermore, similarly to Figure 3, it is shown that when the average transmit power constraint is considered, the performance is maximized when time splitting is used. Note that as it has already been shown in the analysis, the optimal value of θ for the density splitting protocols does not depend on the density of RRHs. However, this is not the case for the values of τ that maximize the performance of the time splitting-based protocols. To this end, the optimal value of τ for the case of peak power constraints is illustrated in Figure 8, from which it becomes apparent that after a certain value of λ, its further increase has a minor impact on the optimal value of τ, which is due to the saturation of the ergodic rate with respect to the RRHs’ density.

## 7. Conclusions

In this paper, new strategies and policies have been proposed and optimized in order to maximize the ergodic rate in ultra-dense WPNs. More specifically, considering that each of the RRHS can operate either as power beacon or access point, the design strategies aim at balancing the trade-off between the average harvested energy and the average distance between the user and the shortest RRH. All formulated problems have been optimally solved by using convex optimization tools and providing insightful closed-form solutions. The derived solutions offer helpful guidelines for the design of ultra-dense WPNs and, thus, can serve as a useful roadmap for the practical implementation of this framework. The presented numerical results illustrate the performance of the proposed strategies and provide useful remarks regarding their comparison. Among others, it has been shown that using all the RRHs as power beacons for a portion of time (i.e., time splitting) outperforms the continuous utilization of a portion of RRHs as power beacons (i.e., density splitting) when an average power constraint is imposed or when the transmit power of the power beacons and the RRHs density are relatively small. Moreover, it has been illustrated that the performance of hybrid time and density splitting for certain system’s parameters can be achieved by using either time or density splitting.

## Data Availability

Not applicable.
